# Our Journal—Volume Three

**Published:** 1886-03

**Authors:** 


### OUR JOURNAL—VOLUME THREE.

With this number THE ATLANTA MEDICAL AND SURGICAL JOURNAL begins its third year under its new management. The past year has been a successful one for THE JOURNAL. The already large subscription list has steadily increased until we have reached a circulation unheard of in Southern Medical Journalism. For this great success we are indebted to the profession all over the South. Our policy in future will be, as it has been in the past, to promote the honor and welfare of the entire profession, and to this end we invite the assistance of all who will write in the spirit of our motto, "*Pax et scientia sed veritas sine timore.*"

For the satisfaction of advertisers who may desire to avail themselves of our large circulation, we subjoin the following sworn statement of the Superintendent of our publishing house:

FRANKLIN PRINTING HOUSE,
ATLANTA, GA., February 25, 1886.

I certify that the circulation of THE ATLANTA MEDICAL AND SURGICAL JOURNAL is *five thousand* copies, commencing with the new volume, March, 1886, and that I have printed of this number *five thousand and two hundred* copies.

JOHN S. PRATHER, JR., Superintendent.

Sworn to and subscribed before me this February 25, 1886.

THOMAS J. PEEPLES,
[SEAL.] *Notary Public for Fulton County.*

